# Evaluation of hepatic fibrosis with noninvasive methods: transient elastography in HIV infected patients

**DOI:** 10.1186/1471-2334-13-S1-P19

**Published:** 2013-12-16

**Authors:** Elena Dumea, Simona Claudia Cambrea, Maria Margareta Ilie, Lucian Cristian Petcu, Adrian Streinu-Cercel

**Affiliations:** 1 Ovidius University, Constanța, Romania; 2 Clinical Hospital of Infectious Diseases, Constanța, Romania; 3 Carol Davila University of Medicine and Pharmacy, Bucharest, Romania

## Background

Measurement of liver stiffness using transient elastography is a noninvasive, reliable predictor of hepatic fibrosis, but data on its use in HIV infected patients are limited.

## Methods

We evaluated 39 patients with HIV/AIDS infection, who were under monitoring at the HIV Day Clinic of the Clinical Hospital of Infectious Diseases Constanța. In these patients we evaluated liver fibrosis using FibroScan. Statistical analysis was performed with SPSS 17.0.

## Results

The mean FibroScan score was 5.153 kPa, with a standard deviation (SD) of 1.493 kPa.

There were 19 patients with CD4 counts between 200-500 cells/cmm; in this group, the mean value of FibroScan was 5.378±1.415 kPa. In the 20 patients with CD4 count above 500 cells/cmm, the mean FibroScan score was 4.94±1.56 kPa.

There were no statistically significant differences between groups regarding the CD4 count (p=0.36). In this study we did not select cases of patients with severe immunodepression (CD4 count below 200 cells/cmm).

Regarding HIV viral load (VL), we split patients into two groups, above and below 400 copies/mL. There were 31 patients with VL less than 400 copies/mL; the mean FibroScan score was 5.161±1.633 kPa. There were 9 patients with VL more than 400 copies/mL, with a mean FibroScan score of 5.125±0.818 kPa. There were no statistically significant differences between groups regarding HIV VL (p=0.931).

## Conclusion

In the studied patients there were no significant differences in terms of liver fibrosis evaluated by FibroScan. We included no patients with severe immunodepression and all patients received combined antiretroviral therapy.

Noninvasive methods for the evaluation of liver fibrosis are less studied in HIV patients. Further extensive studies are necessary for such patients in order to evaluate cutoffs for assessing liver fibrosis and cirrhosis.

